# Computer‐assisted fetal laser surgery in the treatment of twin‐to‐twin transfusion syndrome: Recent trends and prospects

**DOI:** 10.1002/pd.6225

**Published:** 2022-08-29

**Authors:** Anouk Marlon van der Schot, Esther Sikkel, Marc Erich August Spaanderman, Frank Patrick Hector Achilles Vandenbussche

**Affiliations:** ^1^ Department Obstetrics & Gynecology Radboudumc | Amalia Children's Hospital Nijmegen The Netherlands; ^2^ Department Obstetrics & Gynecology Maastricht UMC+ Maastricht The Netherlands; ^3^ Department Obstetrics & Gynecology Helios Klinikum Duisburg Germany

## Abstract

Fetal laser surgery has emerged as the preferred treatment of twin‐to‐twin transfusion syndrome (TTTS). However, the limited field of view of the fetoscope and the complexity of the procedure make the treatment challenging. Therefore, preoperative planning and intraoperative guidance solutions have been proposed to cope with these challenges. This review uncovers the literature on computer‐assisted software solutions focused on TTTS. These solutions are classified by the pre‐ or intraoperative phase of the procedure and further categorized by discussed hardware and software approaches. In addition, it evaluates the current maturity of technologies by the technology readiness level and enumerates the necessary aspects to bring these new technologies to clinical practice.

## Key points

What's already known about this topic?

- Fetal laser surgery in the treatment of twin‐to‐twin transfusion syndrome is a complex surgery.
- Technological solutions regarding preoperative planning and intraoperative guidance have been proposed to reduce the risk of related complications.

What does this review add?

- This review uncovers the literature on computer‐assisted software solutions.

## 1 INTRODUCTION

Fetal laser surgery has become the treatment of choice for twin‐to‐twin transfusion syndrome (TTTS) stage 2–4 between 16 and 26 weeks.^1^ It directly addresses the underlying placental vascular pathology and restores the vascular balance of both fetuses. Ever since the first fetoscopic laser surgery was reported in 1990,^2^ many different entry techniques, instruments, and access diameters have been described for this procedure.^3^

Even in the most experienced fetal therapy centers, survival rates remain below 90%. The stagnation of survival rates is due to, on the one hand, the fact that, despite single‐port minimally invasive entry, iatrogenic preterm premature rupture of the membranes (iPPROM) still does occur. On the other hand, it is because the limited field of view of the fetoscope hampers a complete overview. This limited view, together with the complexity of the surgical task of recognizing the vascular equator midst the very variable topography of chorionic vessels, can result in incomplete surgery, leading to recurring or reversal of the TTTS. It is widely agreed upon that the two critical points during laser surgery are first, determination of the ideal entry point's location, and, secondly, and even more importantly, fast and accurate recognition of the anastomotic vessels, especially for trainees and fetal surgeons with limited experience.^4^

Unlike in almost every other field, the placental vascular topography is unique and unpredictable, and even the most experienced fetal surgeons must first map out the vascular anatomy for every case anew. Technological solutions regarding the optimization of planning and performing fetal surgery have been proposed to cope with these challenges. First, preoperative planning and simulation provide a better preoperative understanding of the patient‐specific placental anatomy. Second, a computer‐assisted guidance system may help the surgeon in the intraoperative scenario by enabling quick, accurate, and complete placental anastomoses detection.

Despite the high potential of these techniques, introduction into clinical practice has been limited. This review uncovers the literature on preoperative planning and intraoperative guidance in treating TTTS focused on computer‐assisted software solutions. Besides, it evaluates the current state by the technology readiness level (TRL) and enumerates the necessary aspects to bring these new technologies into clinical practice.

## 2 METHODS

### 2.1 Search strategy

The databases PubMed and Web of Science were electronically searched up to October 2021 for publications at any time to identify eligible studies. The search strategy consisted of index terms and keywords related to preoperative planning and computer‐assisted techniques (image processing, computer‐assisted, 3D reconstruction, surgical navigation, surgical planning, and related terms) combined with medical terms regarding laser surgery in the treatment of TTTS (twin‐to‐twin transfusion syndrome, fetal surgery, and related terms). The titles and abstracts of the found literature were screened for relevance, and full‐text copies of the selected articles were retrieved and read in full. Reference lists were hand‐searched for additional literature to ensure that all relevant studies were included in the search results.

### 2.2 Study selection

Studies were included if they:

- were written in the English language;
- reported original data;
- were published in peer‐reviewed journals;
- were written with a primary focus on the purpose of preoperative planning or intraoperative guidance in the treatment of TTTS.

Studies were excluded if they:

- focused solely on the diagnosis of TTTS;
- focused solely on the training of the surgical procedure in the treatment of TTTS.

### 2.3 Study classification

Table 1 summarizes the purpose, anatomical structures of interest, and requirements in the preoperative and intraoperative phases.^4^, ^5^ Furthermore, the studies are categorized by the different steps in these phases; (1) imaging, (2) classification, (3) segmentation, (4) reconstruction, and (5) simulation.^6^ In this review, image segmentation refers to the technical process of (semi)automatical extraction of a region of interest by dividing the image into segments based on a specified description, such as vasculature detection. The image segments help in reducing the complexity of the fetoscopic image to simplify further processing.

**TABLE 1:** Overview of the purpose, anatomical structures of interest, and preoperative planning and intraoperative guidance requirements, based on^4^, ^5^

Purpose	Anatomical structures of interest	Requirements
Preoperative planning		
Determining the optimal entry point Simulating fetoscopic trajectory	Placenta Fetuses Umbilical cord insertions Vascular equator	Safe for fetus and mother Immediate result _____________________ • Cornerstone technique is ultrasound
Intraoperative guidance		
Providing better visualization of placental surface Optimizing navigation and orientation	Placental surface Vascular equator Singular, superficial anastomoses Inter‐fetal membrane	Real‐time guidance No interruption with current clinical workflow _____________________ • Cornerstone technique is fetoscopy

#### 2.3.1 Preoperative phase

The primary interest in the preoperative phase is to aid in the determination of the optimal entry point and simulate the surgical procedure. Relevant anatomical structures in this phase are the location of the placenta, umbilical cord insertions, and fetuses, as well as an indication of the location of the vascular equator.^4^ The preoperative phase consists of the diagnostic process, ultrasound examination, counseling, and preparation of the operation room.^4^ Once fetal laser surgery is indicated, surgical preparation time is limited to approximately 24 h. This review searched for studies that meet the requirements, such as enhanced visualization of the placental vascularity, 3D Power Doppler, tomographic ultrasound imaging, and magnetic resonance imaging (MRI).

#### 2.3.2 Intraoperative phase

The primary interest in the intraoperative phase is to guide a fast and accurate identification of the anastomoses. Relevant anatomical structures in this phase are the placental anastomoses on the equator and the inter‐fetus membrane for reference. This phase consists of the insertion of the surgical instruments, orientation and mapping of the placental surface, and laser coagulation of the anastomoses.^4^ Ideally, surgery time should be kept to a minimum, as well as laser time and energy.^7^, ^8^ This review searched for studies about the enhancement of visualization, artificial expansion of the field of view, and robotic assistance during the procedure, among others.

### 2.4 Evaluation of techniques

#### 2.4.1 Technology readiness level

**FIGURE 1:**
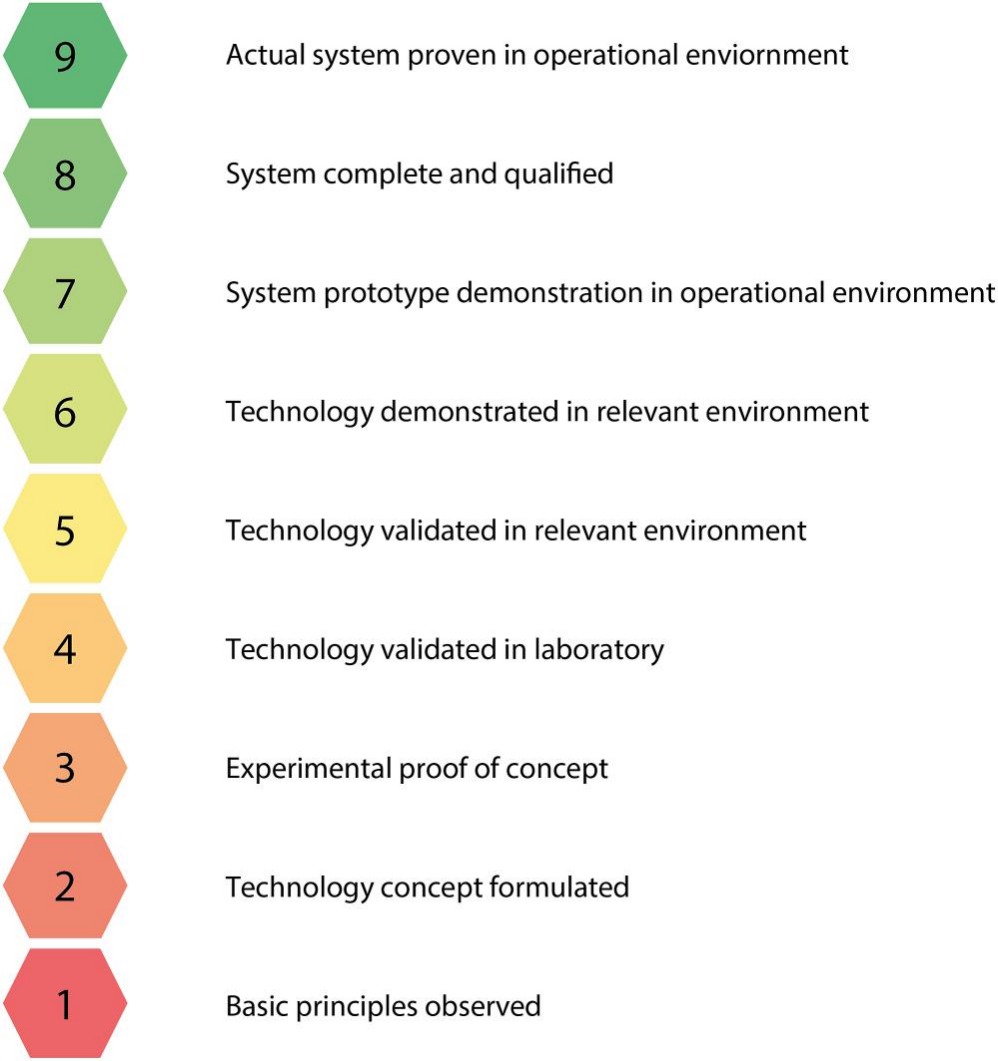
Technology readiness level (TRL) used to classify the maturity of the discussed technological solutions

The TRL is often used to rate techniques.^9^ The National Aeroneutics and Space Administration presented this measure to provide a systematic measurement of technological maturity, categorizing technologies from a basic level to the state of commercial deployment. We adapted this nine‐level TRL scale for clinical evaluation (Figure 1) and categorized the techniques as follows. The highest levels were used for the most mature technologies, for example, commercially available (TRL 9) or incorporated into a commercial design (TRL 8). In the evaluation, functional systems validated in clinical (TRL 7) and preclinical (TRL 6) studies are considered system prototypes. Validation of the prototype in a suitable environment (TRL 5) was classified when the technique is tested with fetal surgical images; there is minimum interference with surgical routine; the method works in the timeframe of the surgical procedure, or expert users' experiences were positively evaluated. Validation in a laboratory setting (TRL 4) and experimental proof of concept (TRL 3) were used for techniques tested with ex‐vivo or less realistic data. The formulation of technology concepts (TRL 2) and observation of basic principles (TRL 1) was classified with the lowest TRLs.

#### 2.4.2 F1‐score

**FIGURE 2:**
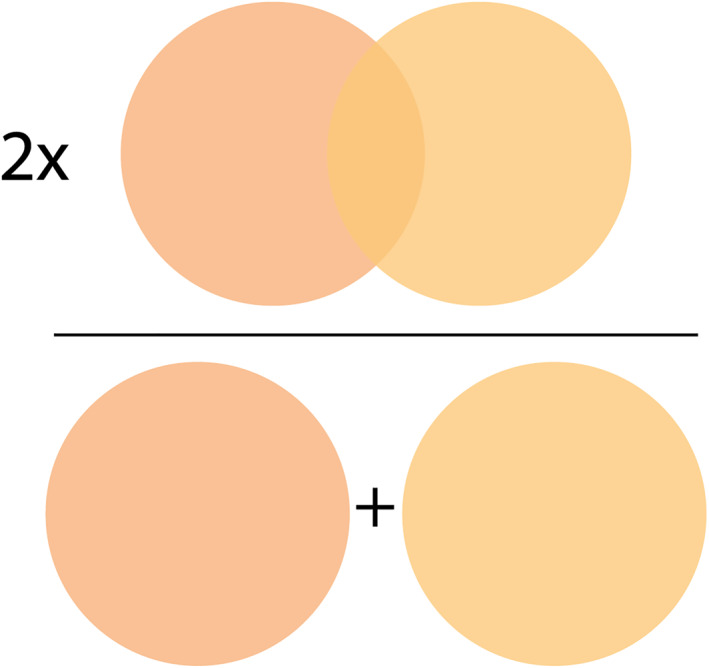
Visualization of the F1‐score, also known as Dice similarity coefficient, equals twice the number of common pixels to both images divided by the sum of the number of pixels in each image

In the analysis of binary classification and segmentation, many authors used the F1‐score to measure the method's accuracy. The F1‐score is calculated by two times the area of overlap in pixels, divided by the total number of pixels in both images, see Figure 2.^10^, ^11^ The score ranges between 0 and 1, representing zero and perfect overlap, respectively.

## 3 RESULTS

**FIGURE 3:**
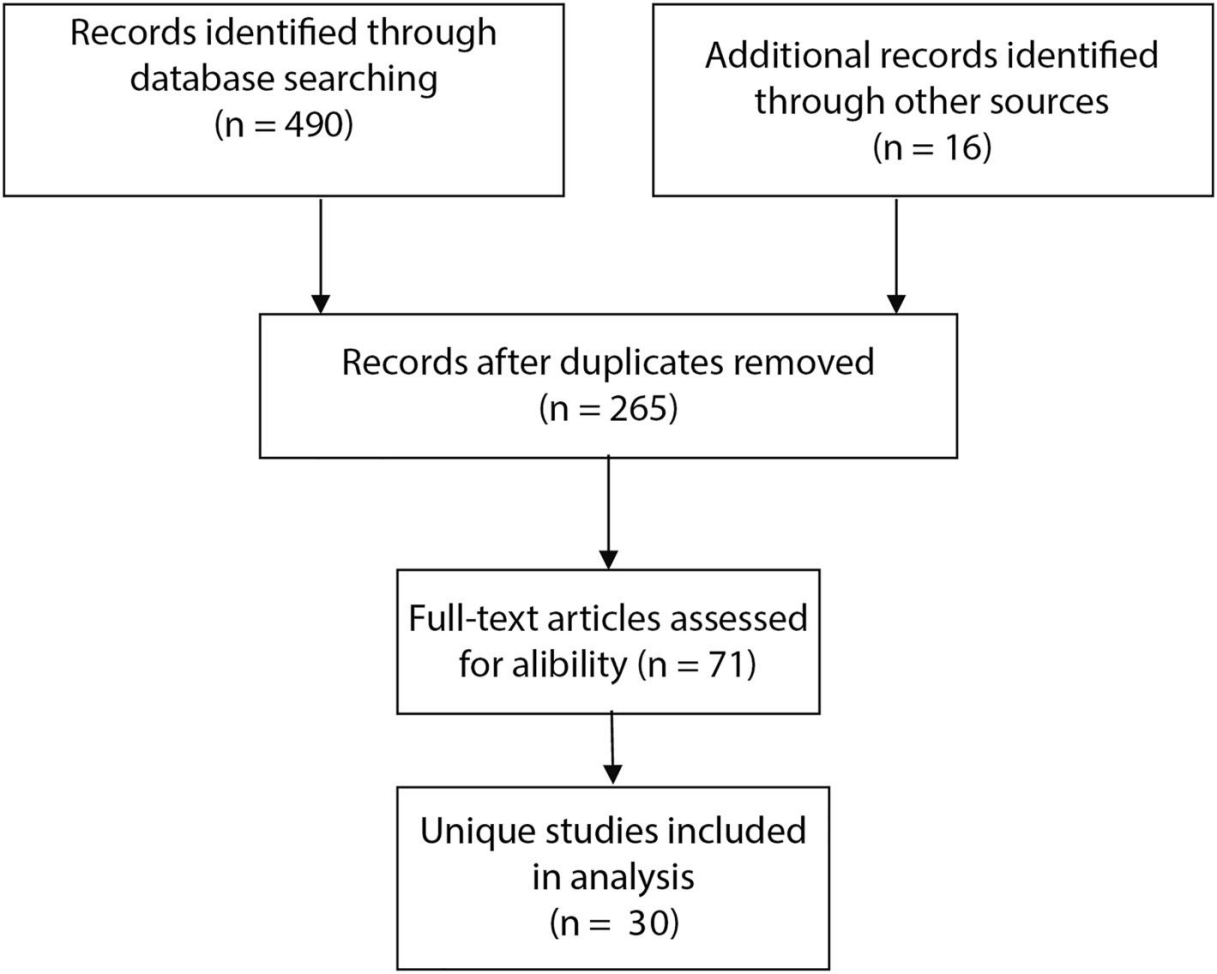
Preferred Reporting Items for Systematic Reviews and Meta‐Analylsis (PRISMA) flow diagram for the different phases of this review

Figure 3 shows the flow diagram of our literature search results. All studies that matched our inclusion criteria were included. A total of 265 unique studies were identified, of which 194 were excluded after reviewing the title and abstract. The remaining 71 were read in full. Subsequently, 40 studies were excluded for being out of scope or meeting the exclusion criteria, resulting in 30 studies being included in the analysis.

**FIGURE 4:**
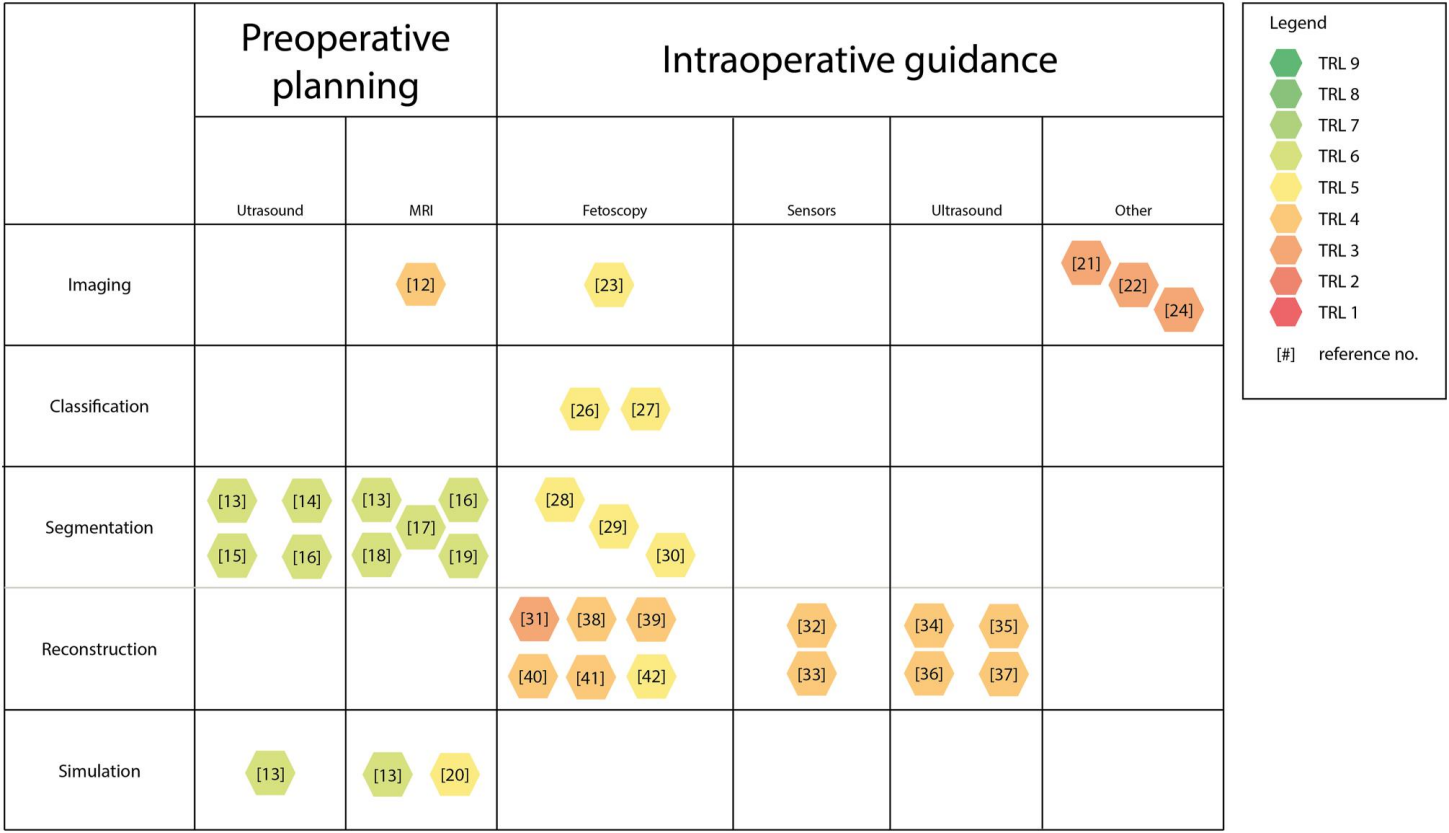
Overview of the discussed research, categorized by surgical phase. Technology readiness level (TRL) is indicated by color according to Figure 1

Figure 4 describes the relationship between the surgical phase, hardware, and software of the articles in this section. According to the TRL, a color indication, as in Figure 1, is given of the current state of these research articles. In the following paragraphs, the reviewed articles are explored and discussed. More details on each paper can be found in the appendix (Table A1–A7).

### 3.1 Preoperative planning

Table 2 summarizes the discussed research in the preoperative phase and provides an overview of each technique's potential impact and limitations.

**TABLE 2:** A summary of the discussed research regarding preoperative planning, including the potential impact and limitations

Reference	Method	Potential impact	Limitations
Imaging			
^12^	Visualization of the placental vasculature in a virtual reality environment	Better preoperative anatomical understanding	Need of a virtual reality environment
			MRI data
Segmentation (US)			
^13^, ^14^, ^15^, ^16^	Fully automatic segmentation of placenta (relatively high F1‐score) and umbilical cord detection	Important component of a framework for preoperative planning, including 3D reconstruction and simulation	Relatively low F1‐score for vasculature segmentation
Segmentation (MRI)			
^13^, ^16^, ^17^, ^18^, ^19^	Fully automatic segmentation of intrauterine environment, including uterus, placenta, and its vasculature	Important component of a framework for preoperative planning, including 3D reconstruction and simulation	MRI data
Simulation			
^20^	3D reconstruction of patient‐specific anatomical structures	Better preoperative understanding of intrauterine environment, including placenta, fetus, and uterus	No vasculature reconstruction of and umbilical cord
			MRI data
			Relative long computational time
^13^	A patient‐specific preoperative planning and simulation platform includes segmentation and registration of the maternal soft tissue, uterus, umbilical cord insertions, placenta, and vasculature.	Better preoperative understanding of intrauterine environment, including placenta, vasculature, umbilical cord insertions	MRI data
		Simulation of patient‐specific fetal laser surgery to determine the optimal site of insertion and reproduce optimal trajectory of the fetoscope to reach the vascular equator	
		Fast enough for clinical implementation	

#### 3.1.1 Imaging

Improvement of the visualization of placental vessels using a virtual reality (VR) environment has been proposed in a case study by Novotny et al.^12^ In their study, medical professionals were able to interactively identify potential anastomoses using MRI data directly from volume visualizations in the VR visualization. Qualitative feedback suggests that the VR visualization is easy to understand and allows intuitive data exploration, but the method suffers from the fact that they have only been tested for one singleton pregnancy (TRL 4). In addition, the method relies on the availability of a VR system, which makes clinical implementation in most fetal therapy centers challenging.

#### 3.1.2 Segmentation

We selected studies regarding segmentation in the preoperative phase. Four of them used ultrasound data and five used MRI data. All methods have been demonstrated using in vivo data, which gives a TRL of 6. Ultrasound segmentation has mainly focused on the segmentation of the placenta and its vasculature, using 3D and 4D ultrasound imaging.^13^, ^14^, ^15^, ^16^ With MRI data, more structures have been segmented, for example, soft tissue, the intrauterine cavity, and the fetal brain.^17^

The reported mean F1‐score between the ground truth data and placental segmentation results using ultrasound data is slightly higher than the F1‐score for MRI data, respectively, 0.79 and 0.75.^13^, ^14^, ^15^, ^16^, ^17^, ^18^, ^19^ It is the other way around for vasculature segmentation, where ultrasound had a lower mean F1‐score than MRI, respectively, 0.65 and 0.81.^13^, ^14^, ^15^, ^16^, ^18^ In our method, we also selected the umbilical cord as an important anatomical structure; however, not many authors have focused on umbilical cord segmentation. Using ultrasound data, Torrents‐Barrena et al. and Perera‐Bel et al. described the localization of the umbilical cord insertions with a detection rate of 85% and 40% in twin pregnancies, respectively.^14^, ^15^ They did not report an F1‐score. A mean F1‐score of 0.77 has been reached using MRI data for umbilical cord segmentation.^16^ Together, these results indicate that MRI improves the segmentation results, especially for vasculature and umbilical cord segmentation.

#### 3.1.3 Simulation

In 2001, Luks et al. were one of the first to use 3D reconstructions from MRI images. They described two cases of TTTS for preoperative planning (TRL 5), enabling virtual surgical navigation in fetal surgery.^20^ Virtual models of the fetuses, placenta, and uterus created a virtual environment to simulate the anatomy, locate the intertwin membrane's location, and plan the best entry point and the fetoscope's angle. With their visibility study, they showed that preoperative planning and virtual surgical navigation in fetal surgery are possible, although attempts to visualize the anastomoses and the vascular equator were unsuccessful, and the computational time was relatively long (1–2 h).

In 2019, Torrents‐Barrena et al. proposed the first planning and simulation system for fetal laser surgery in the treatment of TTTS.^13^ They included the segmentation methods for soft maternal tissue, uterus, umbilical cord insertions, placenta, and vasculature for the ultrasound and MRI using deep learning techniques. A full exploration of the relevant intrauterine environment in a simulated environment was possible by creating 3D models by combining MRI and ultrasound. Their platform was assessed by clinicians and independent users and highly appreciated, and the developed system does not compromise the operation time (TRL 6). For clinical implementation, the techniques require further (clinical) validation. However, they presented a potential tool to be implemented in real and complex TTTS surgeries, also for experienced surgeons.

### 3.2 Intraoperative guidance

Table 3 summarizes the discussed research in the intraoperative phase and provides an overview of each technique's potential impact and limitations.

**TABLE 3:** A summary of the discussed research regarding intraoperative guidance, including the potential impact and limitations. iPPROM = iatrogenic preterm premature rupture of the membranes

Author (year)	Method	Potential impact	Limitations
Imaging			
^21^, ^22^	Indocyanine green (ICG) fluoroscopy	Better visualization of the placental vasculature	Risk of feto‐maternal use of ICG is unknown
^23^	Real‐time computerized enhancement of fetoscopic video frames	Ease the visibility of the fetoscopic images intraoperatively	Fixed set of chosen parameters
		Important component of a framework for intraoperative guidance	
^24^	Integration of optical ultrasound	Better visualization of the placental vasculature and accurately depth reconstruction	Synchronization is needed between different modalities
			Large diameter instruments
Classification			
^26^, ^27^	Automatic detection of valid fetoscopic frames	Important component of a framework for intraoperative guidance	Limited (annotated) fetoscopic database available
Segmentation			
^28^, ^29^, ^30^	Automatic segmentation of the placenta, its vasculature, and the inter‐fetal membrane	Important component of a framework for intraoperative guidance	Limited (annotated) fetoscopic database available
			Integration with the classification method is necessary to select suitable frames
Reconstruction			
^31^	Planar feature‐based fetoscopic image registration	*Increased field of view for fast and accurate detection of anastomoses.	Long computational time
			Performs worse in low structured images and less and poor visibility
			Phantom and ex vivo data
^32^, ^33^	Integration of electromagnetic trackers in combination with visual data	Reduction of accumulated drift during video mosaicking	Use of external sensors hinder clinical implementation
^34^, ^35^, ^36^, ^37^	Mapping of fetoscopic images onto ultrasound image‐constructed 3D model	Added value due to 3D perspective of the patient's anatomy	Calibration between US model and fetoscopic images hinders clinical implementation
		Can handle dynamic changes in uterus	Large diameter instruments, resulting in increased risk of iPPROM
^38^	Intensity‐based registration	Works better for images with low texture and poor illumination	Computational heavy
		Image registration based on stable features	Accumulation of error (drift)
^39^, ^41^	Deep learning methods for fetoscopic image registration using in‐vivo videos	Fast and accurate reconstruction	Accumulation of error (drift)
			Limited fetoscopic images available
			Lack of ground truth
^42^	SLAM framework	Increased field of view	No relocalization
		Loop closure	Camera calibration not resolved
			Cannot handle dynamic changes in environment

#### 3.2.1 Imaging

Several approaches to guide surgeons and improve intrauterine visualization have been proposed. Early research discussed fluorescence fetoscopy to enhance the visualization of placental vessels in an intrauterine environment.^21^, ^22^ Further research has focused on the digital enhancement of fetoscopic images to facilitate placental blood vessel identification.^23^ This real‐time computerized enhancement can directly guide the surgeon intraoperatively and has been tested with in vivo fetoscopic images (TRL 5). In addition, it could be of value in a larger framework for intraoperative guidance, including surface reconstruction. Another approach uses optical ultrasound combined with fetoscopic images and robotic control (TRL 3).^24^ These modalities can provide an increased field of view, yield additional information below the visible tissue surface, or enable visualization in challenging intrauterine situations. However, large diameter instruments are currently required, hindering clinical implementation due to an increased risk of iPPROM.^25^

#### 3.2.2 Classification

Classification methods are an essential component of the framework for intraoperative guidance. In this perspective, Bano et al. introduced a multiple‐class classification method to automatically detect frames with a clear view, occlusion, tool, and ablation.^26^ This method resulted in an F1‐score of 0.85 for a clear view and 0.74 for occlusion, using in vivo images (TRL 5). In addition, Vasconcelos et al. proposed an automatic binary classification method to detect laser ablation frames using in vivo images resulting in an average F1‐score of 0.86 (TRL 5).^27^ These methods for automatic detection of valid frames can be of significant value in the following segmentation and reconstruction steps.

#### 3.2.3 Segmentation

Scanning and visualizing the placental vasculature is of primary interest during fetoscopic laser surgery, including the anastomoses and the inter‐fetal membrane.^4^ Segmentation images can be of added value in the visualization, as well as for the reconstruction and simulation process. Casella et al. focused on the automatic segmentation of the inter‐fetal membrane, reporting an F1‐score of 0.88 (TRL 5).^28^, ^29^ In addition, Sadda et al. have investigated the automatic segmentation of vessels in vivo fetoscopic images (TRL 5). Their segmentation results were used for visual enhancement during surgery and reported an F1‐score of 0.55.^30^

#### 3.2.4 Reconstruction

Early research into fetoscopic image reconstruction has focused on classical image feature‐based registration methods from planar placental images. Reeff et al. investigated feature‐based methods for fetoscopic mosaicking using ex vivo placenta data. They showed that it is possible to match around 40 frames successfully (TRL 3).^31^ It took 60 min to build a mosaic of only 20% of the placental surface.^31^ Frames with shallow structural content were especially challenging, making clinical implementation far away, but their method generated noticeable results and provided the basics for other research.

The classic image registration algorithms have the disadvantage of error accumulation, that is small errors in the relative transformations will accumulate over time. This accumulation of error is also known as drift. Methods using a fusion of the fetoscopic images and an electromagnetic tracker are proposed to minimize this drifting, resulting in a robust mosaic of 366 frames of an ex vivo placenta (TRL 4).^32^, ^33^ Another way to overcome drift is by using an ultrasound image‐based method for rigid fetoscope localization, as introduced by Yang et al. By mapping fetoscopic views to a 3D placental phantom model acquired through ultrasound, they could use preoperative data and bridge existing gaps in placental imaging.^34^, ^35^, ^36^, ^37^ However, these methods have only been tested using synthetic, phantom, and ex vivo human placentas, which is not comparable to the in vivo situation (TRL 4). Besides, the current clinical workflow and regulations hinder trackers and additional hardware in the intraoperative setting.

Most improvements have been achieved with direct image registration methods,^38^ including pixel‐wise gradient alignment, deep learning methods, and registration of segmented placental vessels (TRL 4 and 5).^39^, ^40^, ^41^ More recently, Li et al. proposed a simultaneous localization and mapping technique that creates or updates a map of the unknown intrauterine environment while simultaneously keeping track of the fetoscopic camera. They combine vessel segmentation with global consistency optimization to minimize accumulative drift errors (TRL 5).^42^ By automatically recognizing when the fetoscope has returned to a previously mapped region on the placenta, the accumulation of error can be corrected, making this solution suitable for long‐range overlapping fetoscopic sequences.

## 4 DISCUSSION

This review identified 30 articles related to computer‐assisted fetal laser surgery in treating TTTS. Current trends and challenges are reflected, and the results are subjectively evaluated. In addition, the TRL was used to indicate the technological maturity of the techniques. Initial steps in the development of computer‐assisted preoperative planning and intraoperative guidance dealing with the critical points during the laser procedure, that is, determining the entry point's location and rapid recognition of the anastomoses, are discussed. Since survival rates increase with growing operator experience, the discussed techniques might be especially beneficial for trainees and fetal surgeons with limited experience. It should be clear, though, that the discussed techniques are not yet implemented in the clinical setting. For a swift translation of computer‐assisted techniques into clinical practice, it is important to have an infrastructure where clinical, scientific, engineering, and regulatory expertise are combined early in the process.

Altogether, it can be concluded that preoperative planning for fetal laser surgery in the treatment of TTTS is closer to clinical implementation than intraoperative guidance. In this preoperative phase, computer vision and deep learning techniques enable the integration of segmentation and simulation algorithms that can facilitate the surgeon in the determination of the optimal site of insertion and reproduce the optimal trajectory of the fetoscope to reach the vascular equator prior to surgery. Noteworthy is that fetal MRI has gained much attention in the literature as a valuable tool to visualize placental vessels in this application. A major advantage of US remains its availability. However, there still may be a future for MRI in the preoperative setting. Fetal MRI is relatively complete, fast, and close to clinical implementation and especially in simulation settings, MRI might be beneficial.^13^

Also, intraoperative guidance can aid the surgeon in fast and accurate identification of the anastomoses and thereby decrease the mental load. Larger diameter fetoscopes that would expand the field of view should not be used because they increase the risk of iPPROM to unacceptably high levels. Remarkably, many authors focused on the real‐time reconstruction method to expand the field of view of the fetoscope. For fast clinical implementation, it is important to focus on relatively low computational time, high accuracy, the use of in vivo data, and a reduction of the accumulation of drift error. In addition, a relocalization module, multimapping, or dynamic method will help if tracking is lost due to rapid fetoscopic movements or fetal parts moving to the field of view. Finally, it was remarkable that most researchers did not take the fetoscopic camera's optical distortions into account, while approaches for fluid‐immersed fetoscopic camera calibration have been proposed.^43^, ^44^

Difficulties arise, however, when an attempt is made to implement the techniques in clinical practice. In addition, a lack of ground truth and big datasets may hinder the fast development of deep learning techniques. Initial steps in public databases on which every research group can compare their results and may provide a solution have been taken.^45^, ^46^ Long‐term directions should focus on integrating preoperative planning, simulation, and intraoperative guidance to help the fetal surgeon during the treatment of TTTS. From a (trainee) fetal surgeon's view, the future ‘ideal’ pre‐and intraoperative framework is an intelligent system that requires minimum interference with surgical routine, is easy to handle, and is universally applicable. This system will contain preoperative data to simulate the surgery and determine the best entry point, as well as real‐time guidance that can provide an increasingly panoramic view of the anastomoses. In the meantime, it is essential to focus on the needs and expectations of the fetal surgeon. Instead of optimizing technical innovations, research should deliver value to the clinical setting and support fetal surgeons with current challenges. A map of the placental vasculature with acceptable accuracy may already contribute to a better understanding of the interoperative scene and reduce the risk of incomplete surgery.

## 5 CONCLUSION

In conclusion, this review provides an overview of the recent technological developments in preoperative planning and simulation and intraoperative guidance in treating TTTS, where preoperative planning is closer to clinical implementation than intraoperative guidance. The critical technologies for future development are artificial intelligence and computer vision methods. Ideally, long‐term directions should focus on integrating preoperative planning and intraoperative reconstruction. The main goal should be to deliver value to the surgeon by artificially expanding the field of view of small‐diameter fetoscopes to reduce complications like premature rupture of the membranes and incomplete surgery.

## CONFLICT OF INTEREST

The authors have no conflicts of interest to declare.

## Supporting information

Supplementary Material

## Data Availability

The data that support the findings of this study are available on request from the corresponding author.
