# Supplementary material for: Computer‐assisted fetal laser surgery in the treatment of twin‐to‐twin transfusion syndrome: Recent trends and prospects
Source: Prenat Diagn. 2022 Aug 29;42(10):1225–34. doi: 10.1002/pd.6225 (PMC9541851; doi:10.1002/pd.6225)
Supplement: Supplementary file 1 — Supplementary Material [file PD-42-1225-s001.docx]

Table A1. Overview of the articles about imaging in the preoperative phase. GW = gestational weeks. ﻿ICG = indocyanine green.

| Author (year) | Anatomical structure | Image modality | Automatic/ manual | Data | Results | Computational time |
| --- | --- | --- | --- | --- | --- | --- |
| Novotny et al. (2020)^12^ | Vessels | VR + MRI | Manually | MRI of singleton fetus (GW 25) | - Identification of blood vessels of 1mm - Precision > 59.8% - Sensitivity > 38.3 % | - <557 s   (visualization) |

Table A2. Overview of the articles about segmentation in the preoperative phase. DC = dichorionic, GW = gestational weeks, MC = monochorionic. * estimated from figure

| Author (year) | Anatomical structure | Image modality | Automatic/manual | Data | Results | | |
| --- | --- | --- | --- | --- | --- | --- | --- |
| Ultrasound | | | | | **Placenta (F1-score)** | **Vasculature (F1-score)** | **Umbilical cord (detection rate)** |
| Torrents-Barrena et al. (2019)^13^ | Placenta, vasculature | 3D ultrasound + Doppler | Semi- automatic | - 21 patients - (MC) twin pregnancies - 17-33 GW | - 0.72 ± 0.11 | - 0.69 ± 0.08 | N/A |
| Torrents-Barrena et al. (2020)^14^ | Placenta, vasculature, umbilical cord | 3D HD -flow ultrasound | Fully automatic | - 70 patients - Twin pregnancies - 17-37 GW | - 0.82 ± 0.13 | - 0.72 ± 0.09 | - 85% |
| Perera et al. (2020)^15^ | Placenta, vasculature, umbilical cord insertion | Doppler ultrasound | Semi-automatic | - 29 patients - Singleton (24) and MC (5) pregnancies | - ≥ 83.0 ± 5.4 (singleton pregnancies) - ≥ 83.1 ± 4.6 (MC pregnancies) | - ≥ 57.6 ± 18.7 (singleton pregnancies) - ≥ 56.2 ± 10.3 (MC pregnancies) | - 75% (singleton pregnancies) - 40% (MC pregnancies) |
| Torrents-Barrena et al. (2021)^16^ | Placenta | 3D ultrasound | Fully automatic | - 60 patients - Singleton and (MC/DC) twin pregnancies - 17-37 GW | - 0.76 ± 0.12 | N/A | N/A |
| MRI | | | | | **Placenta (F1-score)** | **Vasculature (F1-score)** | **Umbilical cord (F1-score)** |
| Wang et al. (2015) ^19^ | Placenta | MRI | Semi-automatic | - 6 patients - Singleton pregnancies - First trimester | ~ 0.84 ± 0.10** | N/A | N/A |
| Torrents-Barrena et al. (2019)^13^ | Soft tissue, uterus, placenta, vasculature, umbilical cord | 1.5T MRI | Semi-automatic | - 21 patients - (MC) twin pregnancies - 17-33 GW | 0.72 ± 0.10 | 0.81 ± 0.13 | 0.73 ± 0.11 |
| Torrents-Barrena et al. (2020)^18^ | Intra-uterine cavity | 1.5T MRI | Fully automatic | - 71 patients - Singleton and MC twin pregnancies | N/A | N/A | N/A |
| Badaoui et al. (2020)^17^ | Maternal skin, pelvis, maternal spine, maternal bladder, uterus, placenta | 1.5T MRI | Semi-automatic | - 3 patients - Singleton pregnancies - 28-34 GW | Not reported | N/A | N/A |
| Torrents-Barrena et al. (2021)^16^ | Uterus, umbilical cord, placenta, brain, lungs | 1.5T MRI | Fully automatic | - 60 patients - Singleton and (MC/DC) twin pregnancies - 17-37 GW | 0.68 ± 0.13 | N/A | 0.81 ± 0.07 |

Table A3. Overview of the articles about simulation in the preoperative phase. MC = monochorionic

| Author (year) | Anatomical structure | Image modality | Automatic/manual | Data | Quantitative results | Computational time (min) |
| --- | --- | --- | --- | --- | --- | --- |
| Luks et al. (2001)^20^ | Fetuses, placenta, and uterus | 1.5T MRI | Semi-automatic | - 2 cases of TTTS with anterior placenta | Not reported | 60-120 |
| Torrents-Barrena et al (2019)^13^ | Soft tissue of the mother, the uterus, umbilical cords, placenta and its vascular tree | 3D US + 1.5T MRI | Automatic (manual editions possible) | - 18 MC twin pregnancies   - 7 amniotic fluid discordance   - 8 TTTS   - 3 non-pathological twin pregnancies (GW 17–33) | Segmentation results can be found in **Table A2**. | 45-55 |

Table A4. Overview of the articles about imaging in the intraoperative phase. fps = frames per second

| Author (year) | Image modality | Automatic/manual | Data | Quantitative results | Computational time |
| --- | --- | --- | --- | --- | --- |
| Harada et al. (2009)^21^ | ICG fluoroscopy | Automatic | - Monkey and pregnant rats | - Clear detection the vascular network | ﻿30–60 seconds after intravenous administration |
| Ishiyama et al. (2011)^22^ | ICG fluoroscopy | Automatic | - Pregnant rabbits | - ﻿ Detection 0.2 mm diameter vessels | ﻿15 s after ICG injection for about 10min |
| Sadda et al. (2019)^23^ | Fetoscopic images | Automatic | - 10 patients - 189 frames | - Identifying marker on a blood vessel   - Enhanced video frames accuracy 74.27% (SE 0.97)   - Unenhanced video frames accuracy 63.78% (SE 2.79) | 24.58 fps |
| Dwyer et al. (2019)^24^ | Robotic fetoscope with optical ultrasound and stereo camera | Semi-automatic | - Phantom data | - Surface visualization of 80mm x 80mm - Repeatability of 0.446 ± 0.139 and 0.267 ± 0.017 mm for a raster and spiral scan respectively | 43 – 45 min scanning time |

Table A5. Overview of the articles classification in the intraoperative phase.

| Author (year) | Anatomical structure | Image modality | Automatic / manual | Data | Results (F1-score) |
| --- | --- | --- | --- | --- | --- |
| Vasconcelos et al. (2018)^27^ | Ablation | Fetoscopic images | Fully automatic | - 5 patients - 49,527 frames | Average: 0.86 |
| Bano et al. (2020)^26^ | Clear view, occlusion, tool, ablation | Fetoscopic images | Fully automatic | - 7 patients - 69,390 frames | - Average: 0.89 - Clear view: 0.85 - Occlusion: 0.74 |

Table A6. Overview of the articles about segmentation in the intraoperative phase.

| Author (year) | Anatomical structure | Image modality | Automatic / manual | Data | Results (F1-score) | |
| --- | --- | --- | --- | --- | --- | --- |
|  |  |  |  |  | **Vessels** | **Inter-fetal membrane** |
| Casella et al. (2020)^28^ | Inter-fetal membrane | Fetoscopic images | Fully automatic | - 6 patients - 150 frames | N/A | 0.92 |
| Casella et al. (2021)^29^ | Inter-fetal membrane | Fetoscopic images | Fully automatic | - 20 patients - 2000 frames | N/A | 0.88 ± 0.14 |
| Sadda et al. (2019)^30^ | Vessels | Fetoscopic images | Fully automatic | - 10 patients - 345 frames | 0.55 ± 0.22 | N/A |

Table A7. Overview of the articles about reconstruction in the intraoperative phase. EM = electromagnetic.

| Author (year) | Method | Automatic / manual | Data | Results (quantitative) | Computational time |
| --- | --- | --- | --- | --- | --- |
| Feature-based image registration | | | | | |
| Reeff et al. (2006)^31^ | Feature based | Fully automatic | - Ex-vivo setup in water | • Successful matched frames: 33/40  • Precision: 67%   - Mean recall: 25.5% | - 60 minutes to build mosaic |
| External sensors | | | | | |
| Tella-Amo et al. (2018)^32^ | EM tracker in combination with visual data | Fully automatic | - Synthetic data - Phantom placenta - Ex-vivo human placenta | - Matched frames   - Synthetic**:** 3770   - Phantom: 902   - Ex-vivo: 366 | - Synthetic**:** 52.95 s - Phantom**:** 558.04 s |
| Tella-Amo et al. (2019)^33^ | EM tracker in combination with visual data and pose optimization | Semi-automatic | - 273 frames synthetic - 701 frames phantom | - Mean residual error: ~7 after 12 seconds (syn) - Mean residual error: ~25 after 20 seconds (phantom) |  |
| Ultrasound imaging | | | | | |
| Yang et al. (2015)^34^ | Fetoscopic images aligned with 3D ultrasound model | Semi-automatic | - Monkey placenta - Phantom placenta - 2x 100 frame sequence | - Image overlay error (80 frames): 2.38 mm (std 1.88 mm) - Successfully matched: > 500 frames | - 6 min (500 frames) |
| Yang et al. (2015)^37^ | Fetoscopic images aligned with 3D ultrasound model | Semi-automatic | - Monkey placenta - Phantom placenta | - Error: 0.2mm, 1° (20 frames) - Error: 4.61 mm over a total displacement of 45 mm | - Tracking: 0.75 s - Initialization: 2.23 s |
| Yang et al. (2016)^35^ | Fetoscopic images with vision-based tracking | Semi-automatic | - Monkey placenta - Phantom placenta | - RMS error: 5.2 % | - Not reported |
| Yang et al (2016)^36^ | Fetoscopic images | Semi-automatic | - Monkey placenta - Phantom placenta | - ﻿Successful matched frames: 282/300 | - ﻿3.33 fps |
| Direct image registration | | | | | |
| Peter et al. (2018)^38^ | Direct alignment of image gradient orientations | Fully automatic | - In-vivo fetoscopic video - 600 frames | - Correct registration: 79.6% - Doubtful registration: 11.2% - Incorrect registration: 9.2% | - 180 minutes to build mosaic |
| Bano et al. (2020)^39^ | Direct intensity-based registration using vessel segmentation maps | Fully automatic | - 6 patients - 483 frames | - 400 matched frames - Structural similarity index: ~0.9 (0.75-0.98) - Intersection over union: ~0.475 (0.39-0.50) | - Not reported |
| Deep learning methods | | | | | |
| Gaisser et al. (2018)^40^ | Extracting matchable features | Fully automatic | - 745 images - Ex-vivo placenta, yellow and green-turbid liquid | - 100% registration - 98% (yellow) - 91% (green) | Not reported |
| Ahmad et al. (2020)^41^ | Estimate placenta pose | Fully automatic | - Simulated data - Silicon phantom placenta | - 87% accuracy for simulated dataset - 81% accuracy for phantom dataset | - 250 fps |
| SLAM | | | | | |
| Li et al. (2021)^42^ | SLAM | Fully automatic | - Simulation data - 6 In-vivo video sequences | Not reported | Not reported |
